# Boron‐embedded multifunctional nanoplatform toward potential combined boron neutron capture therapy and immunomodulation in glioma

**DOI:** 10.1002/smo2.70107

**Published:** 2026-09-16

**Authors:** Yiming Liu, Xuejian Wang, Bangjian Li, Kun Shao, Jianlong Su, Yang Wang, Lin Cheng, Zhihao Wu, JinRong Li, Guanyi Zhang, Xu Wang, Yuxuan Zhang, Yuanhao Liu, Guangzhe Li

**Affiliations:** ^1^ Department of Pharmaceutical Engineering State Key Laboratory of Fine Chemicals School of Chemical Engineering Dalian University of Technology Dalian China; ^2^ State Key Laboratory of Fine Chemicals Frontiers Science Center for Smart Materials Oriented Chemical Engineering School of Chemical Engineering Dalian University of Technology Dalian China; ^3^ Department of Urology First Affiliated Hospital of Dalian Medical University Dalian China; ^4^ Neuboron Medtech Ltd. Nanjing China

**Keywords:** boron neutron capture therapy, covalent organic frameworks, glioblastoma, real‐time tracking, tumor microenvironment

## Abstract

Glioma poses a formidable therapeutic challenge due to its cellular heterogeneity, invasiveness and the protective blood‐brain barrier (BBB). Boron neutron capture therapy represents a promising binary targeted radiotherapy, but its efficacy is hampered by insufficient tumor‐specific accumulation, limited real‐time monitoring capabilities of boron agents, and the immunosuppressive tumor microenvironment (TME). Although L‐4‐boronophenylalanine (BPA) is the only boron agent approved in Japan for Boron neutron capture therapy, its suboptimal tumor specificity and rapid metabolism limit effective accumulation in glioma. In this work, we engineered a theranostic strategy based on a brain‐targeting nanoplatform (DM&Ce6@COF‐Ang). Boron‐embedded APTES‐COF‐1, with a high intrinsic boron content of 13.5%, served as both a therapeutic boron agent and a nanocarrier to deliver the STING agonist DMXAA (abbreviated as DM) and the fluorescent probe chlorin *e*6 (Ce6). The targeting ligand Angiopep‐2 was utilized to facilitate low‐density lipoprotein‐related protein‐1 (lipoprotein receptor‐related protein 1) receptor‐mediated transcytosis across the BBB. Specifically, DM@COF‐Ang demonstrated superior tumor‐specific accumulation in orthotopic glioma‐bearing mice, reaching a tumor boron concentration of 11.3 ± 2.9 ppm (natural abundance boron, ∼19.8% ^10^B) (T/N ≈ 4.0), whereas BPA yielded only 1.9–3.8 ppm (T/N ≈ 2.0). Simultaneously, the nanoplatform selectively delivered DMXAA to glioma, triggering STING pathway activation and reprogramming the immunosuppressive TME. Importantly, the co‐delivered Ce6 functioned as a modal imaging agent, enabling accurate tracking of DM@COF‐Ang biodistribution and confirming the feasibility of real‐time monitoring. This multifunctional nanoplatform achieved synergistic tumor‐specific boron delivery, real‐time pharmacokinetic monitoring, and immunomodulation. The modular design establishes a versatile foundation for developing personalized, potential BNCT‐based combination therapies.

## Graphical abstract

A brain‐targeting multifunctional nanoplatform (DM&Ce6@COF‐Ang) was engineered by employing boron‐enriched APTES‐COF‐1 as both the therapeutic boron agent and the nanocarrier co‐delivery of DMXAA and Ce6. This nanoplatform achieved synergistic tumor‐specific boron delivery, real‐time pharmacokinetic monitoring, and immunomodulation. The modular design establishes a versatile foundation for developing personalized, BNCT‐based combination therapies.

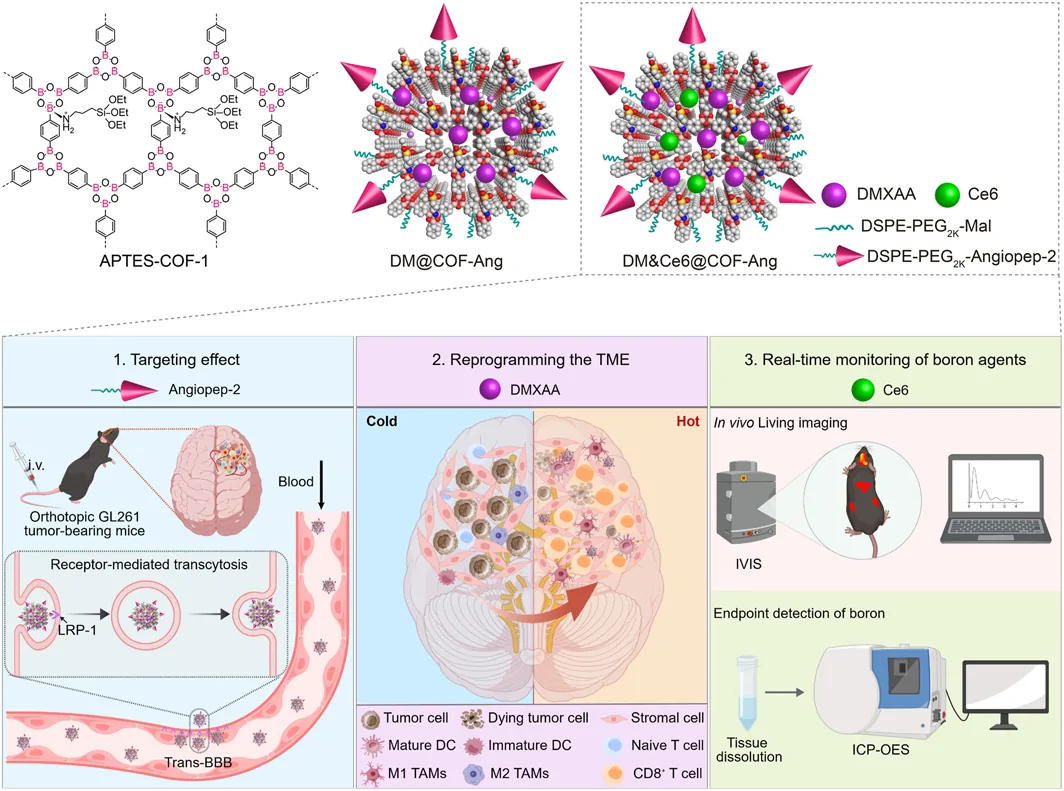

## 1 INTRODUCTION

Glioma is a highly malignant brain tumor characterized by rapid proliferation and invasion growth, posing a significant global health burden.[^1^, ^2^] Current clinical management of glioma primarily involves surgery, radiotherapy and chemotherapy.[^3^, ^4^] However, complete surgical excision is rarely feasible due to the ambiguous boundaries of glioma.[^5^, ^6^] The standard treatment regimen consists of maximal safe resection followed by radiotherapy and concomitant chemotherapy with temozolomide.^[7]^ Nevertheless, the non‐selectivity of these modalities inevitably results in damage to both neoplastic and healthy tissues, and fails to eradicate microinvasive tumor cells that confers only modest clinical benefit with a median survival time of just 15 months.[^8^, ^9^] Thus, non‐invasive therapeutic strategies that can selectively eliminate tumor cells without damaging the surrounding brain tissue are needed to improve outcomes in glioma treatment.

Boron neutron capture therapy (BNCT) is a non‐invasive binary radiotherapy modality that employs a thermal neutron beam and boron‐10 (^10^B)‐containing agents to selectively eradicate tumor cells.^[10]^ This approach relies on thermal neutrons inducing nuclear capture reactions with ^10^B, generating high linear energy transfer particles (^4^He particles and ^7^Li recoil nuclei) with a path length restricted to 4.5–10 μm. This range approximates the diameter of a single cell, ensuring that the cytotoxic effects are strictly confined to the ^10^B‐enriched tumor cells with minimal impact on adjacent normal cells.[^11^, ^12^] It is worth noting that clinical trials of BNCT have shown notable efficacy to treat locally aggressive tumors, including glioma, melanoma, and recurrent unresectable head and neck cancer.[^13^, ^14^] The first clinical application of BNCT in glioma patients was pioneered by Sweet et al. in the 1950s.^[15]^ Despite decades of development, so far only L‐4‐boronophenylalanine (BPA) and sodium borocaptate (BSH) have been used clinically, with only BPA being approved in Japan for unresectable, locally advanced, or recurrent head and neck cancers.[^2^, ^16^, ^17^] Currently, multiple injectable formulations of Borofalan (^10^BPA) have been approved for clinical trials in China.

Insufficient boron enrichment within the tumor region remains a major obstacle to BNCT efficacy. BPA is taken up by cells via the L‐type amino acid transporter 1 (LAT1), an amino acid transporter overexpressed in cancer cells.[^18^, ^19^] Nevertheless, its accumulation in glioma remains suboptimal due to inadequate tumor‐targeting specificity, rapid metabolism and blood‐brain barrier (BBB) restriction.[^12^, ^20^, ^21^] An ideal boron delivery agent should meet the following conditions: (i) high tumor uptake (≥20 μg ^10^B per 10^9^ cells in vitro or gram of tissue in vivo); (ii) tumor‐specific accumulation, requiring tumor to normal tissue (T/N) and tumor to blood (T/B) boron concentration ratios above 3:1; (iii) low cytotoxicity when in the absence of neutron irradiation.^[22]^ Besides, to provide a scientific basis for precise patient selection and the design of personalized treatment regimens, the concurrent development of companion diagnostics for therapeutic boron drugs is also essential.^[23]^ For example, fluorine‐18‐labeled BPA (^18^F‐BPA) has been utilized in clinics, which exhibits similar biodistribution dynamics with BPA,^[24]^ enabling real‐time drug tracing via positron emission tomography (PET). Evidently, the development of boron delivery agents should prioritize both highly efficient tumor accumulation and real‐time boron monitoring to achieve theranostic integration.^[23]^

Covalent organic frameworks (COFs) have emerged as attractive drug‐delivery carriers due to their high surface area, tunable porosity, and functionalizability.[^25^, ^26^] COFs have been utilized to deliver various therapeutic agents such as doxorubicin,^[27]^ docetaxel,^[28]^ and Toll‐like receptor 7 agonist imiquimod.^[13]^ COF‐1, one of the first COFs ever reported, was originally constructed from 1,4‐benzenediboronic acid (BDBA) through boroxine linkages.^[29]^ To improve the structure stability against hydrolysis, a dative boron‐nitrogen bond (B–N) was formed via introducing 3‐aminopropyl triethoxysilane (APTES) into COF‐1, yielding an amine‐functionalized COF‐1 (APTES‐COF‐1).[^25^, ^30^] Given the abundance of intrinsic B–O–B linkages within its framework, APTES‐COF‐1 possesses 2.6‐fold higher boron content relative to BPA (13.5% vs. 5.2%), representing a boron‐enriched nanomaterial with significant potential for boron delivery. Meanwhile, its well‐defined pore architecture provides additional advantages for surface functionalization and the co‐delivery of diverse therapeutic molecules and/or imaging agents.^[25]^

Angiopep‐2 is a 19‐amino‐acid peptide that binds to the low‐density lipoprotein receptor‐related protein 1 (LRP‐1).^[31]^ LRP‐1 is widely expressed in the central nervous system (CNS), with its expression particularly upregulated in pathological foci such as malignant astrocytoma, especially glioma.^[32]^ By facilitating receptor‐mediated transcytosis (RMT), Angiopep‐2 enables therapeutics to cross the BBB and selectively accumulate at brain tumor sites.^[33]^ Although other LRP‐1‐targeting peptides (e.g., L57, M1, RAP12) have been identified, Angiopep‐2 remains the most extensively studied and commonly utilized ligand for glioma delivery.[^34^, ^35^, ^36^, ^37^] Many clinical studies have demonstrated the effectiveness of Angiopep‐2‐conjugated paclitaxel (ANG1005) in patients with breast cancer with leptomeningeal carcinomatosis and recurrent brain metastases.[^38^, ^39^] Furthermore, nanocarriers conjugated with Angiopep‐2 have been shown to significantly prolong the systemic circulation of drugs and enhance their accumulation in brain tumors.[^40^, ^41^, ^42^, ^43^, ^44^]

In addition to direct cytotoxicity, BNCT has been reported to induce abscopal effects, which are associated with immune activation.[^13^, ^14^, ^45^] However, glioma is characterized as an immunologically “cold” tumor with an extremely complex and highly immunosuppressive tumor microenvironment (TME).^[46]^ In the TME of glioma, immunosuppressive tumor‐associated microglia and macrophages (TAMs) are predominated with a high association with poor prognosis.[^47^, ^48^] The presence of these immunosuppressors inevitably compromises the BNCT‐induced immune responses, consequently fostering immune tolerance.[^49^, ^50^] DMXAA, a potent stimulator of interferon genes (STING), triggers anti‐tumor immune responses by promoting dendritic cell (DC) maturation and reshaping the TME.[^51^, ^52^] It has been reported that the combination with DMXAA can significantly suppress the pro‐tumoral M2 phenotype of tumor‐associated macrophages (TAMs), thereby promoting their repolarization towards the anti‐tumoral M1 phenotype.^[53]^ By reversing immunosuppression and converting gliomas into “hot” tumors, DMXAA may therefore prime the TME for BNCT to elicit robust antitumor immune responses.

**SCHEME 1:**
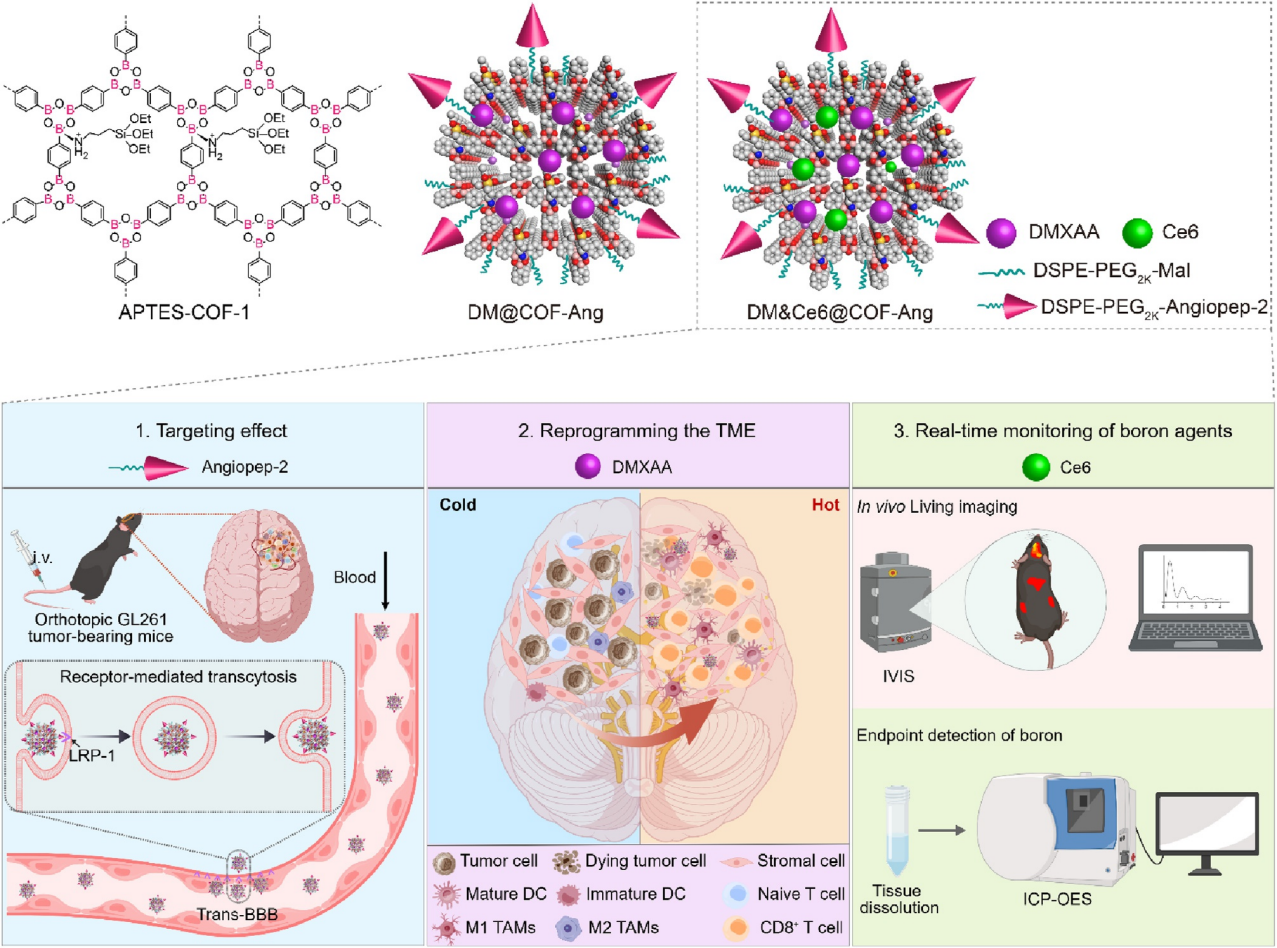
Schematic illustration of the structure and mechanism of action of the nanoboron agent.

In this study, we developed an Angiopep‐2 functionalized, glioma‐targeting nanoboron multifunctional platform (DM&Ce6@COF‐Ang) utilizing APTES‐COF‐1 as both the therapeutic boron agent (natural abundance boron, ∼19.8% ^10^B) and the nanocarrier co‐delivery of immunomodulator DMXAA and fluorescent probe, Chlorin *e6* (Ce6) (Scheme 1). Angiopep‐2 was employed to facilitate the transport of the nanocomposites across the BBB via RMT through the LRP‐1, thereby enabling selective accumulation of the cargoes in glioma. Specifically, the results of in vivo biodistribution demonstrated that DM@COF‐Ang achieved superior tumor targeting compared to BPA, with a T/N ratio of approximately 4.0, and a peak accumulation of 11.3 ± 2.9 μg boron/g tissue (ppm) at 2 h post‐injection. Concurrently, the nanoboron agent selectively delivered DMXAA to the tumor site, triggering STING pathway activation, which in turn reversed the immunosuppressive TME, and elicited potent tumor‐specific systemic immune responses. More importantly, real‐time fluorescence imaging confirmed that the co‐delivered Ce6 effectively probed the biodistribution of DM@COF‐Ang, confirming its reliability for real‐time tracking. Taken together, this work established an integrated theranostic strategy for potential BNCT. By co‐incorporating the fluorescent probe (Ce6) and the immune adjuvant (DMXAA) into a boron‐embedded COF‐based nanocarrier (APTES‐COF‐1) with Angiopep‐2 functionalized surface, we developed an integrated platform that synergistically achieved tumor‐specific boron delivery, real‐time dynamic monitoring, and local immune modulation in glioma. This integrated strategy not only propels future personalized BNCT but also establishes a versatile framework for developing multimodal combination therapies.

## 2 MATERIALS AND METHODS

### 2.1 Materials

1,4‐benzenediboronic acid (BDBA), (3‐Aminopropyl) triethoxysilane (APTES), Vadimezan (DMXAA), and 5,5′‐dithiobis‐(2‐nitrobenzoic acid) (DTNB) were purchased from Aladdin Chemical Reagent Co., Ltd. (Shanghai, China). Mesitylene and 1,4‐dioxane were purchased from Energy Chemical (Shanghai, China). Distearoyl phosphatidylethanolamine‐methoxy polyethylene glycol (DSPE‐mPEG_2k_) and DSPE‐PEG_2k_‐maleimide (DSPE‐PEG_2k_‐Mal) were purchased from Pengshuo Biotechnology Co., Ltd. Cys‐Angiopep 2 (TFFYGGSRGKRNNFKTEEYC) was purchased from Apeptide Co., Ltd. Chlorin e6 (Ce6) and Potassium dihydrogen phosphate were purchased from Macklin Inc. Methylthiazolyldiphenyl‐tetrazolium bromide (MTT), Diamidino‐2‐phenylindole (DAPI), lipopolysaccharide (LPS), fluorescein sodium, red blood cell lysis solution, and 4% paraformaldehyde were purchased from Beyotime Biotech. Inc.

### 2.2 Synthesis of APTES‐COF‐1

APTES‐COF‐1 was prepared following a literature procedure.^[30]^ In a dry N_2_ environment, 0.165 g 1,4‐benzenediboronic acid (BDBA) was mixed with 0.05 g (3‐Aminopropyl) triethoxysilane (APTES) in 5 mL of a 1:1 v/v solution of mesitylene:1,4‐dioxane. The reaction mixture was sealed with dry N_2_, degassed by three freeze‐pump‐thaw cycles and treated with an ultrasonic water bath for 60 min. The reaction vessel was evacuated, and then the solution was heated to 75°C for 20 h. A white powder was obtained after filtration in atmospheric conditions, and was subsequently dried under vacuum at 120°C. Products were stored at room temperature.

### 2.3 Synthesis of and characterization DSPE‐PEG_2k_‐Ang

The DSPE‐PEG_2k_‐Ang was obtained by the coupling reaction between the maleimide at the end of DSPE‐PEG_2k_‐Mal and the sulfhydryl group at the end of Cys‐Angiopep‐2 in PBS buffer. The reaction was carried out by mixing 3 mg of DSPE‐PEG_2k_‐Mal and 1 mg of Cys‐Angiopep‐2, adding 5 mL of PBS (pH 7.24) and stirring at room temperature for 12 h. The reaction was purified by dialysis, freeze‐dried and stored at −20°C. At different time points, the linkage rates of Cys‐Angiopep‐2 and DSPE‐PEG_2k_‐Mal were calculated by measuring the absorbance of 412 nm of the reaction solution with DTNB. ^1^H nuclear magnetic resonance (NMR) spectra of DSPE‐PEG_2k_‐Ang and DSPE‐PEG_2k_‐Mal were obtained on a Bruker Avance NEO 600M NMR Spectroscopy (Bruker, Germany).

### 2.4 Preparation of boron‐rich nanoboron agent

A mixture consisting of 10 mg APTES‐COF‐1, 2 mg DMXAA, 5 mg DSPE‐PEG_2k_‐Ang, and 58 mg DSPE‐mPEG_2k_ was dispersed in 1 mL of 1,4‐dioxane under continuous stirring at room temperature for 20 min. Afterwards, 9 mL of deionized water was added dropwise within 3 h. After ultrasonication in a water for another 2 h, residual DMXAA, DSPE‐mPEG_2k_ and 1,4‐dioxane were removed through dialysis (MWCO 3500 Da) against deionized water. The purified DMXAA@APTES‐COF‐1‐DSPE‐PEG_2k_‐Angiopep‐2 (DM@COF‐Ang) was freeze‐dried and preserved at −20°C for long‐term storage. Accordingly, APTES‐COF‐1‐DSPE‐mPEG_2k_ (COF), DMXAA@APTES‐COF‐1‐DSPE‐mPEG_2k_ (DM@COF), DMXAA&Ce6@APTES‐COF‐1‐DSPE‐mPEG_2k_ (DM&Ce6@COF), and DMXAA&Ce6@APTES‐COF‐1‐DSPE‐PEG_2k_‐Angiopep‐2 (DM&Ce6@COF‐Ang) were synthesized.

### 2.5 Characterization

Transmission electron microscope (FEI Tecnai G2 F30 STWIN, USA) images were observed at an acceleration voltage of 200 kV, which was equipped with energy‐dispersive X‐ray spectroscopy (EDS). X‐ray photoelectron spectroscopy (Thermo, ESCALAB250Xi, USA) was performed to determine the elemental type and valence states of the components of DM&Ce6@COF‐Ang. APTES‐COF‐1 was tested for crystal structure with X‐ray diffraction (XRD) (SmartLab, Japan). Solid multi‐quantum magic‐angle spinning NMR (Multiple quantum magic angle spinning NMR) spectra were recorded on a DD2‐500 MHz NMR spectrometer (Agilent, USA). Dynamic light scattering measurements of the nanoparticles were conducted using a Zetasizer Nano ZS (Malvern Instruments, Malvern, UK). The purity of the nanoboron agent was assessed using a Sephadex G‐25 gel chromatography column. Thermo‐gravimetric analysis (METTLER, SDTA851e, USA) of the nanoboron agent was performed in the temperature range of 50–500°C. Ultraviolet‐visible (UV‐Vis) absorption spectroscopy was recorded by a Lambda 750S spectrometer (PerkinElmer, USA). The fluorescence spectrum was recorded by a F7000 spectrometer (HITACHI, Japan). The thickness dimension of DM&Ce6@COF‐Ang was obtained by atomic force microscopy (Bruker Dimension Icon, USA). Confocal laser scanning microscopy (FV1000, Olympus Corporation, Japan) was applied for acquiring the confocal images. Inductively coupled plasma optical emission spectrometry (ICP‐OES) (AVIO 500, PerkinElmer, USA) was conducted for quantitative analysis of the content of Boron element.

### 2.6 In vitro boron release assay

A dialysis method was employed to evaluate the in vitro release profiles of boron from the BSH and DM&Ce6@COF‐Ang nanoboron agent. Briefly, 4 mL of BSH and DM&Ce6@COF‐Ang nanoboron agent containing an identical boron concentration was loaded into separate dialysis bags (MWCO, 3500 Da). The sealed dialysis bags were then immersed in PBS supplemented with 56% (v/v) fetal bovine serum (FBS), maintained under gentle shaking at 37°C. At predetermined time intervals, aliquots of the external release medium were collected. The released boron content in the collected samples was quantified by ICP‐OES.

### 2.7 Cell culture

Mouse glioma cell line (GL261) and mouse brain microvascular endothelial cell line (bEnd.3) were purchased from iCell Bioscience Inc (Shanghai, China). GL261 cells were cultured in Dulbecco's modified Eagle's medium (DMEM) supplemented with 10% FBS, 1% penicillin and streptomycin (P/S), 1% 4‐(2‐hydroxyethyl)‐1‐piperazineethanesulfonic acid (HEPES) 1 M Buffer solution and 1% GlutaMAX‐1 glutamine, whereas bEnd.3 cells were cultured in DMEM medium with 1.5 g/L NaHCO_3_, supplemented with 10% FBS, 1% P/S. The culture condition was maintained at 37°C in a 5% CO_2_ atmosphere. All experiments were performed on cells in the logarithmic phase of growth.

### 2.8 Cell uptake

The cell uptake of Ce6, DM&Ce6@COF, and DM&Ce6@COF‐Ang in GL261 cells and bEnd.3 cells after incubation for different time periods was detected by Live Cell Imaging System (DMi8, Leica Microsystems, German). In brief, GL261 cells and bEnd.3 cells were seeded in a 35‐mm‐diameter culture dish at a density of 1 × 10^5^ cells/dish and incubated overnight to allow cell attachment. The medium was then replaced with fresh medium containing Ce6, DM&Ce6@COF, or DM&Ce6@COF‐Ang at a Ce6 concentration of 5 μg/mL. After incubation for 0.5, 1 and 2h as predetermined, the cells were washed thrice with cold PBS and fixed with 4% paraformaldehyde for 30 min. Subsequently, the cell nuclei were labeled with DAPI for 20 min, and the cells were washed three more times with PBS. The images were observed were by a Live Cell Imaging System.

### 2.9 Quantitative analysis of cellular internalization

The GL261 cells (1 × 10^6^ cells per well) were seeded in 6‐well plates and cultured overnight. Covalent organic framework, DM@COF, and DM@COF‐Ang at a boron concentration of 1.2 mM were added to incubate with cells. After incubation for 0.5, 1, 2 and 4 h, the cells were washed three times with PBS and then collected with 0.05% trypsin‐EDTA solution. Subsequently, the collected cell suspension was dissolved in aqua regia for the digestion and the samples were analyzed for boron concentration measurement by ICP‐OES.

### 2.10 In vitro cytotoxicity assay

GL261 cells and bEnd.3 cells were seeded in 96‐well plates at a density of 8 × 10^3^ cells per well and cultured overnight. The cells were then treated with COF, DM@COF, DM&Ce6@COF, or DM&Ce6@COF‐Ang at varying concentrations of DMXAA and incubated for 24 h. After washing, 10 μL of MTT solutions (5 mg/mL) in PBS were added to each well. After incubating the cells for 4 h, the medium was removed carefully, and 150 μL of DMSO was added to each well to dissolve blue formazan. Finally, the absorbance of 490 nm was measured using a microplate reader (Tecan, Austria).

### 2.11 Co‐localization assay

GL261 cells were inoculated in confocal dishes and cultured for 16 h at 37°C. The cells were subjected to different treatments including Ce6,DM&Ce6@COF and DM&Ce6@COF‐Ang, and incubated for 2 h. After washing with PBS, the cells were incubated with LysoTracker (5 μg/mL) in the dark for 30 min at 37°C, washed three more times with PBS, and fixed with 4% paraformaldehyde for 30 min. Then, the cell nuclei were incubated with DIPA for 10 min at room temperature and then washed three more times with PBS. Subsequently, the intracellular fluorescence was observed by confocal microscopy (imaged with a 100× oil‐immersion objective) (Olympus, FV1000, Japan).

### 2.12 Establishment of blood‐brain barrier model

Transwell^®^ inserts (0.4 μm polycarbonate (PC) membranes with a diameter of 6.5 mm) were selected to establish the BBB model in vitro. The bEnd.3 cells (1 × 10^4^ cells per well) were seeded in the apical chamber of the Transwell^®^ and 0.6 mL of DMEM medium was added to the 24‐well plate on the basolateral chamber of the Transwell^®^ for 5 days until the cells were in a confluent state. Add 500 and 200 μL of medium to the apical and basolateral chambers of the Transwell^®^, respectively, at which time make sure the liquid level difference between the two Transwell^®^ chambers was 1 cm. Recorded the liquid level at 1, 4 and 9 h, and observed the changes in the liquid level difference between the two chambers.

The integrality of the in vitro cultured BBB model was investigated by fluorescein sodium permeability. Briefly, fluorescein sodium (50 μg/mL) was added to the apical chamber of the Transwell^®^, and the permeation amount was measured using a multifunctional microplate reader at different time points.

### 2.13 In vitro evaluation of boron‐rich nanoboron agent across the blood‐brain barrier

The GL261 cells were seeded in the basolateral chamber of Transwell^®^ and incubated overnight. The Ce6,DM&Ce6@COF and DM&Ce6@COF‐Ang at Ce6 concentration of 10 μg/mL to the apical chamber of Transwell^®^. After incubation for 3 h, cells were washed with PBS and nuclei were stained with DAPI. Finally, the intracellular fluorescence was observed by a Live Cell Imaging System.

### 2.14 Bone marrow‐derived dendritic cells activation

Immature mouse bone marrow‐derived dendritic cells (BMDCs) (iBMDCs) were isolated from the bone marrow of 8‐week‐old C57BL/6 mice. The iBMDCs seeded in 48‐well plates (2 × 10^5^ cells per well) were incubated with PBS, DMXAA, COF, DM@COF, DM@COF‐Ang, DMXAA + COF at DMXAA concentration of 25 μg/mL for 4 h. After washing with PBS and incubating with fresh medium for 20 h, the maturation status of BMDCs was studied by flow cytometry after staining with anti‐CD11c PE/Cy7, anti‐CD86 PE, and anti‐CD80 antigen presenting cell (APC).

### 2.15 Stimulation and repolarization of bone marrow‐derived macrophages

Bone marrow‐derived macrophages (BMM) from the bone marrow of 8‐week‐old C57BL/6 mice. The BMM seeded in 24‐well plates and then incubated with PBS, DMXAA, COF, DM@COF, DM@COF‐Ang, DMXAA + COF at DMXAA concentration of 25 μg/mL for 48 h. After washing with PBS, the phenotype of BMM was studied by flow cytometry after staining with anti‐F4/80 PE, anti‐CD80 APC, and anti‐CD206 PE/Cy7.

### 2.16 Stimulation and repolarization of primary microglia

Primary Microglia were incubated with DMXAA, COF, DM@COF, DM@COF‐Ang, DMXAA + COF at DMXAA concentration of 25 μg/mL for 48 h at 37°C, respectively. The phenotype of primary Microglia was analyzed through flow cytometry after staining with anti‐CD45‐FITC, anti‐F4/80 PE, anti‐CD80 APC, and anti‐CD206 PE/Cy7.

### 2.17 Orthotopic glioma murine model

C57BL/6j mice aged 6–7 weeks were anesthetized with Avertin (1.25%). A skin incision (∼10 mm) was made from the median frontal to parietal bones. Tumor cell implantation was then performed using a stereotaxic apparatus (RWD Life Science, Inc., Shenzhen, China). Briefly, 2 × 10^5^ GL261 cells in 5 μL PBS were injected into the intracranial cavity of the mouse at 1 mm anterior, 1 mm to the right side of the bregma, and 3 mm deep into the brain. Standard post‐surgery care was given following the protocol approved by the Animal Research Ethics Committee of Dalian University of Technology.

### 2.18 In vivo biodistribution

The orthotopic glioma mice were intravenously injected with free Ce6, DM&Ce6@COF and DM&Ce6@COF‐Ang (Ce6 = 10 mg/kg). At 1, 2, 3, 4 and 6 h after the injection, florescence images were acquired using an in vivo imaging system (IVIS) (Perkin Elmer, USA). At 2 h after the injection, mice were euthanized and the brain, heart, liver, spleen, lung, and kidney were excised for ex vivo imaging.

### 2.19 Pharmacokinetics

The orthotopic glioma mice were intravenously injected with BPA, BSH, DM@COF and DM@COF‐Ang (3.92 mg boron/kg). DMXAA, DM@COF, and DM@COF‐Ang treatments were administered the same dose of DMXAA (15 mg/kg). At the predetermined post‐injection time points, heart, liver, spleen, lung, kidney, brain, and tumor tissues were harvested accordingly. All samples were measured for wet weights immediately after collection and dissolved in aqua regia for the digestion. The determination of tissue boron content in mice by ICP‐OES. Meanwhile, the tumor tissues were taken, weighed and cut, ultrasonicated for 1 h, the supernatant was centrifuged, and the content of DMXAA in the tumor tissues was determined by HPLC (Waters, USA).

### 2.20 Antitumor immunity

The orthotopic glioma mice (day 7 post‐implantation) were randomly divided into 5 groups and separately received the treatments of saline, DMXAA, COF, DM@COF and DM@COF‐Ang. Covalent organic framework, DM@COF, and DM@COF‐Ang were *i*.*v*. administrated at a dose of 3.92 mg boron/kg body weight every 2 days with a total of four doses. DMXAA, DM@COF, and DM@COF‐Ang treatments were administered the same dose of DMXAA (15 mg/kg). All treatments were carried out every 2 days, and a total of four treatments were performed during which the body weights were recorded daily. Three days after the last treatment, the mice were sacrificed. The tumor, spleen, and Tumor‐draining lymph nodes (TDLNs) were collected and subsequently dissociated into single‐cell suspensions. The cells from the TDLNs were stained with anti‐CD11c PE/Cy7, anti‐CD86 APC, anti‐CD80 PE, anti‐CD3 FITC, anti‐CD8 PercP and anti‐CD69 PE for the analysis of DC maturation and T cells activation. The cells from the spleen were stained with anti‐CD3 PE, anti‐CD8 APC, anti‐CD62 L PercP/Cy5.5, anti‐CD3 FITC, anti‐CD8 PercP, anti‐CD4 APC and anti‐CD69 PE for the T cells activation. Tumor cells were stained with anti‐CD11c PE/Cy7, anti‐CD45 FITC, anti‐CD86 APC, anti‐CD80 PE, anti‐F4/80 PE, anti‐CD80 APC, anti‐CD206 PE/Cy7, anti‐CD3 PE, anti‐CD8 FITC and anti‐CD4 APC and then were analyzed by flow cytometry.

### 2.21 Statistical analyses

Processed experimental data were analyzed by GraphPad Prism software using one‐way analysis of variance or Student's *t*‐test. The specific statistical test performed for each experiment was included in the corresponding figure legends. Asterisks represent different levels of significance: *p < 0.05, **p < 0.01, ***p < 0.001, ****p < 0.0001; ns, not significant. All of the flow cytometry analyses were performed using Flow Jo 10.

## 3 RESULTS

### 3.1 Preparation and characterization of nanoboron agent

**FIGURE 1:**
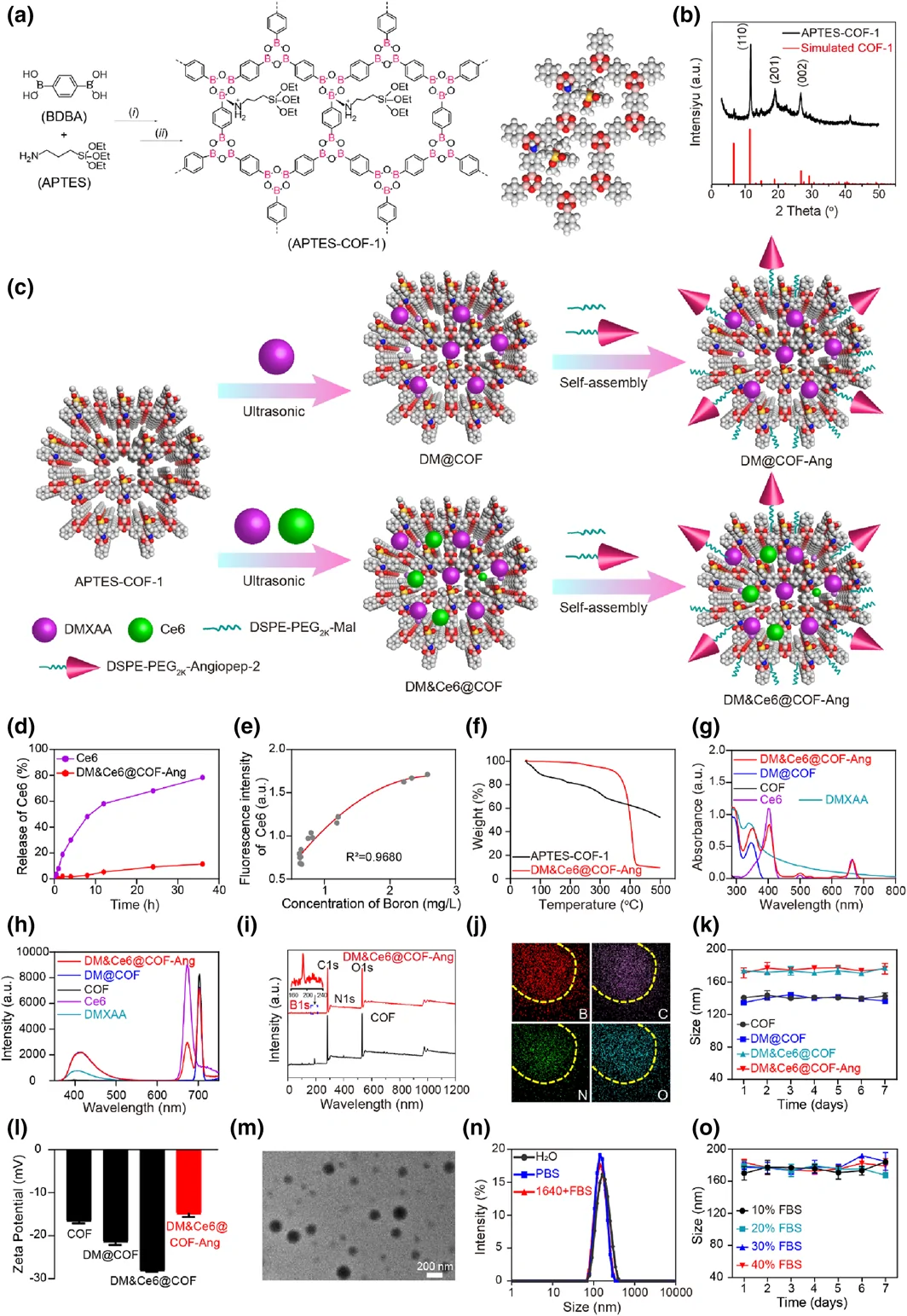
Synthesis and characterization of nanoboron agent. (a) Schematic illustration of synthetic routes of APTES‐COF‐1, yield 97%. *(i)* Three freeze‐pump‐thaw cycles, ultrasonic water bath, 60 min. *(ii)* 75°C, 20 h. (b) Powder X‐ray diffraction (PXRD) patterns of APTES‐COF‐1. (c) Schematic illustration of the preparation of DM@COF‐Ang and DM&Ce6@COF‐Ang nanoboron agent. (d) Time‐dependent Ce6 release profiles of free Ce6 and the DM&Ce6@COF‐Ang nanoboron agent in PBS supplemented with 56% FBS (Mean ± S.D., *n* = 3). (e) Correlation between Ce6 fluorescence intensity and boron concentration measured by ICP‐OES in DM&Ce6@COF‐Ang nanoboron agent. (f) Thermogravimetric analysis (TGA) curves of APTES‐COF‐1 and DM&Ce6@COF‐Ang under air atmosphere. (g, h) UV‐Vis absorbance spectra (g) and fluorescence spectroscopy (h) of free Ce6, DMXAA, COF, DM@COF, and DM&Ce6@COF‐Ang in deionized water. (i) XPS spectrum of COF and DM&Ce6@COF‐Ang. (j) Representative elemental mapping of DM&Ce6@COF‐Ang. Scale bar, 100 nm. (k) Hydrodynamic diameters of COF, DM@COF, DM&Ce6@COF, and DM&Ce6@COF‐Ang in deionized water for 7 days (Mean ± S.D., *n* = 3). (l) Zeta potentials of COF, DM@COF, DM&Ce6@COF, and DM&Ce6@COF‐Ang in deionized water (Mean ± S.D., *n* = 3). (m) TEM images (Scale bar, 200 nm) of DM&Ce6@COF‐Ang nanoboron agent. (n) Hydrodynamic diameters of DM&Ce6@COF‐Ang in different solutions (H_2_O, PBS, or 1640 medium with 10% FBS). (o) Hydrodynamic diameters of the DM&Ce6@COF‐Ang nanoboron agent in 10%–40% FBS for 7 days (Mean ± S.D., *n* = 3). Ce6, chlorin e6; COF, covalent organic framework; FBS, fetal bovine serum; ICP‐OES, inductively coupled plasma optical emission spectrometry; TEM, Transmission electron microscope; XPS, X‐ray photoelectron spectroscopy.

We synthesized an amine‐functionalized APTES‐COF‐1 via a one‐pot reaction using 1,4‐benzenediboronic acid (BDBA) as the structural building block and APTES as both framework stabilizer and Brønsted acid catalyst (Figure 1a). Briefly, BDBA and APTES were dissolved in a 1:1 (v/v) solution of 1,4‐dioxane: mesitylene, followed by degassing through three freeze‐pump‐thaw cycles, sonicating, and stirring at 75°C, resulting in the formation of APTES‐COF‐1 with B‐O‐B linkages. Multiple quantum magic angle spinning of ^11^B revealed tricoordinate boron (B^[3]^) and tetrahedral‐coordinated boron (B^[4]^) species, confirming successful framework formation (Supporting Information S1: Figure S1). The powder X‐ray diffraction pattern of APTES‐COF‐1 showed three major characteristic peaks at 11.6°, 19.2°, and 27.5°, corresponding to the 110, 201, and 002 planes, respectively, demonstrating that the crystal structures of COF‐1 were maintained in APTES‐COF‐1 (Figure 1b).

To enhance aqueous dispersibility and adjuvanticity, a boron‐rich nanoboron agent, DMXAA@APTES‐COF‐1‐DSPE‐mPEG_2k_ (DM@COF) was engineered through hydrophobic‐hydrophobic interaction. Briefly, the DSPE group of DSPE‐mPEG_2k_ was anchored into the hydrophobic channels of APTES‐COF‐1 via hydrophobic interactions, while the STING agonist DMXAA was absorbed onto the surface or within the channels through non‐covalent conjugation. To prepare the targeted nanoboron agent, the DSPE‐PEG_2k_‐Angiopep‐2 (DSPE‐PEG_2k_‐Ang) was synthesized via a click chemistry reaction between the maleimide group of DSPE‐PEG_2k_‐Mal and the thiol group of Cys‐Angiopep‐2. Reaction kinetics, assayed by using Ellman's reagent demonstrated a conjugation efficiency of 92.6% after 12 h (Supporting Information S1: Figure S2). Comparative ^1^H‐NMR analysis revealed the disappearance of the characteristic maleimide peak near 7 ppm in DSPE‐PEG_2k_‐Ang (Supporting Information S1: Figure S3), confirming the complete reaction with the thiol group of Cys‐Angiopep‐2. The purified DSPE‐PEG_2k_‐Ang was incorporated using an identical hydrophobic insertion strategy as DSPE‐mPEG_2k_, yielding DMXAA@APTES‐COF‐1‐DSPE‐PEG_2k_‐Angiopep‐2 (DM@COF‐Ang) (Figure 1c). To enable the traceability of the nanoboron agent, the fluorescent probe Ce6 was co‐encapsulated via hydrophobic‐hydrophobic interaction, resulting in DM&Ce6@COF‐Ang. Post‐synthesis purification via dialysis and dextran gel chromatography confirmed the effective removal of unbound Ce6, as evidenced by the disappearance of the characteristic peak monitored by the UV–vis spectrophotometer (Supporting Information S1: Figure S4). Furthermore, the Ce6 release rate of the DM&Ce6@COF‐Ang nanoboron agent was only 11.43 ± 0.47% after 36 h (Figure 1d). Notably, the fluorescence intensity of Ce6 was positively correlated with the concentration of boron of DM&Ce6@COF‐Ang measured by ICP‐OES (*R* ^2^ = 0.9680) (Figure 1e), demonstrating the feasibility of utilizing Ce6 as a surrogate tracer for tracking the biodistribution of the nanoboron agent.

Thermogravimetric analysis (TGA) demonstrated that DM&Ce6@COF‐Ang exhibited a decomposition temperature of 295°C with a total weight loss of 68.5% at 410°C, confirming the presence of additional components beyond ATPES‐COF‐1 (Figure 1f). UV‐vis absorption and fluorescence spectroscopy of DM&Ce6@COF‐Ang displayed characteristic peaks corresponding to both DMXAA and Ce6, verifying the successful payload loading (Figure 1g,h). Furthermore, elemental composition analysis through X‐ray photoelectron spectroscopy (Figure 1i) and transmission electron microscopy with energy‐dispersive X‐ray spectroscopy (TEM‐EDS) (Figure 1j) confirmed the presence of all expected elements including C, N, O, and B in the nanoboron agent.

Dynamic light scattering revealed that the average dynamic sizes of COF, DM@COF, DM&Ce6@COF, and DM&Ce6@COF‐Ang were 140.73 ± 0.42 nm, 134.57 ± 2.80 nm, 174.07 ± 1.76 nm, and 171.37 ± 6.29 nm, with corresponding zeta potentials of −16.63 ± 0.49 mV, −21.43 ± 0.72 mV, −28.2 ± 0.20 mV, and −14.93 ± 0.72 mV, respectively (Figure 1k,l). Compared to DM&Ce6@COF, the notable increase in the zeta potentials of DM&Ce6@COF‐Ang was attributed to the incorporation of positively charged Cys‐Angiopep‐2. Transmission electron microscope imaging further confirmed that DM&Ce6@COF‐Ang presented a uniform dispersion spherical morphology with sizes ranging 140–180 nm (Figure 1m). Atomic force microscopy revealed a thickness of approximately 5 nm (Supporting Information S1: Figure S5). The leakage of boron from the DM&Ce6@COF‐Ang nanoboron agent over time was monitored by coupled plasma optical emission spectrometry (ICP‐OES). After 24 h of incubation in PBS supplemented with 56% FBS, only 10.54 ± 0.42% of the total boron was detected, demonstrating excellent structural stability in the peripheral blood environment (Supporting Information S1: Figure S6). In the meantime, DM&Ce6@COF‐Ang demonstrated excellent dispersibility in water, PBS and cell culture medium (Figure 1n) and remained stable over 7 days in both aqueous solution and 10%–40% FBS with minimal size variation (Figure 1k,o), indicating its good physiological stability.

### 3.2 Cellular internalization

**FIGURE 2:**
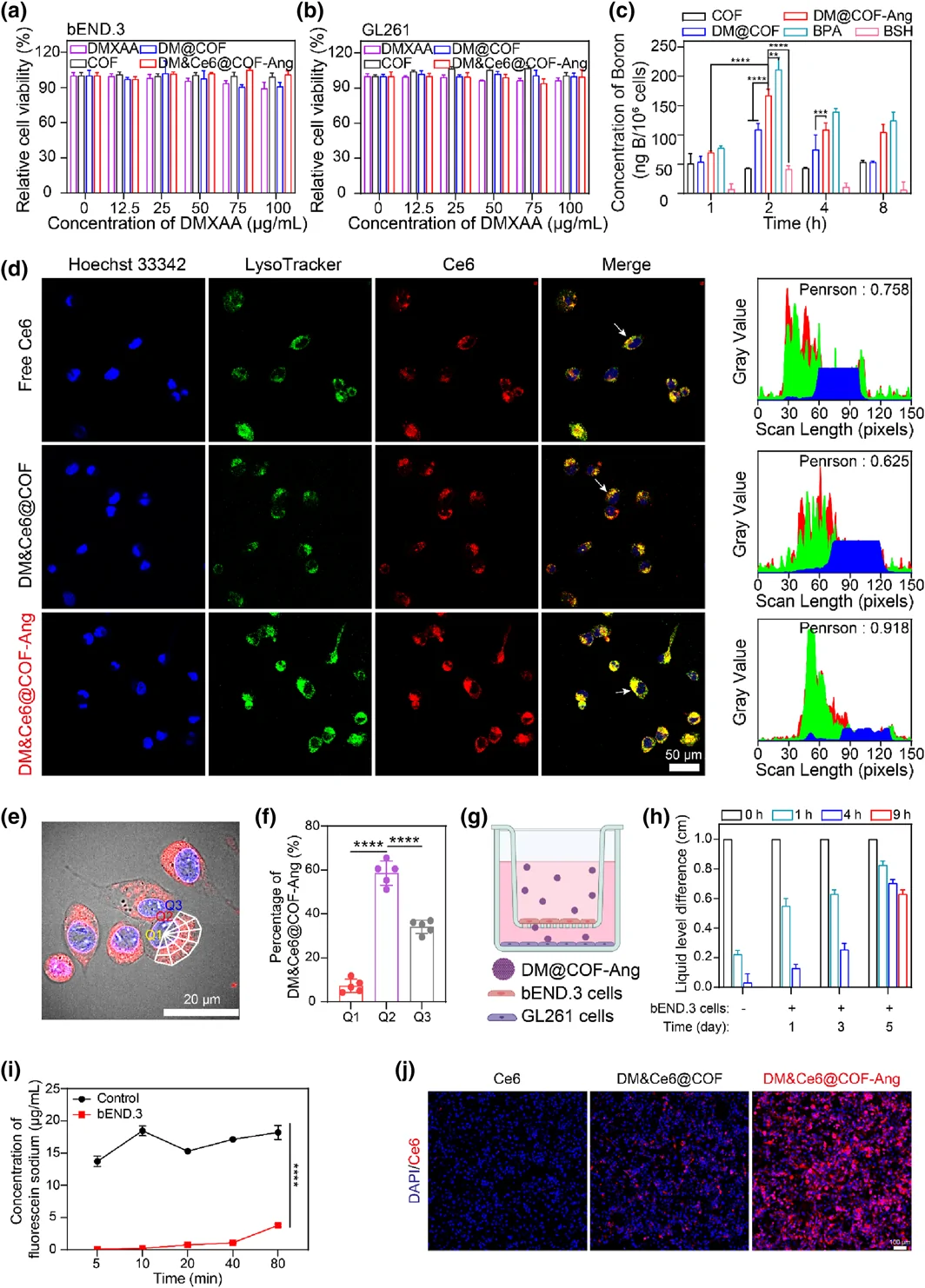
Cellular internalization of nanoboron agent. (a, b) Relative viabilities of bEnd.3 cells (a) and GL261 cells (b) after incubation with DMXAA, COF, DM@COF and DM&Ce6@COF‐Ang at different concentrations (*n* = 4). (c) The boron concentrations in GL261 cells (1 × 10^6^ cells) after co‐incubation with COF, DM@COF, DM@COF‐Ang, BPA and BSH (1.2 mM [B]) for different time points measured by ICP‐OES. (d) Representative images and Pearson correlation coefficient of colocalization with lysosome in GL261 cells treated with free Ce6, DM&Ce6@COF and DM&Ce6@COF‐Ang at a Ce6 concentration of 8 μg/mL for 2 h by confocal microscopy. Scale bar, 50 μm. (e, f) Microarea distributions (imaged with a 100× oil‐immersion objective) (e) and quantitative analysis (f) (*n* = 5) of intracellular DM&Ce6@COF‐Ang after 2 h incubation with GL261 cells. (g) Schematic diagram of in vitro experimental settings to assess the capacity of DM&Ce6@COF‐Ang across BBB. (h) The liquid level difference between two chambers in Transwell^®^ system at different time points after culturing bEnd.3 cells in the apical chamber for 1, 3 and 5 days (*n* = 3). (i) The integrity of the BBB model was evaluated by quantifying fluorescein sodium in the basolateral compartment at different time points (*n* = 3). (j) Representative fluorescence images showing the uptake of Ce6, DM&Ce6@COF, and DM&Ce6@COF‐Ang by GL261 cells located in the basolateral chamber of the BBB model. Scale bar, 100 μm. Data were presented as mean ± SEM. *p*‐values were calculated by using student's *t* test and one‐way analysis of variance (ANOVA). *p < 0.05, **p < 0.01, ***p < 0.001, ****p < 0.0001. BBB, blood‐brain barrier; Ce6, chlorin e6; COF, Covalent organic framework; ICP‐OES, inductively coupled plasma optical emission spectrometry.

The cytotoxicity of nanoboron agent was evaluated in mouse brain microvascular endothelial cells (bEnd.3) and mouse glioma cells (GL261). As shown in Figure 2a,b, DMXAA, COF, DM@COF, and DM&Ce6@COF‐Ang exhibited negligible effects on cell viability on both bEnd.3 and GL261 cell lines, indicating favorable biocompatibility. Cellular uptake efficiency was assessed in GL261 cells as sufficient intracellular boron concentration is essential for effective BNCT. As shown in Supporting Information S1: Figure S7, DM&Ce6@COF‐Ang showed superior internalization at 2 h compared to free Ce6 and non‐targeted DM&Ce6@COF. This enhancement primarily stemmed from the specific binding of Angiopep‐2 to LRP‐1, which is highly expressed on both bEnd.3 and GL261 cells,^[54]^ indicating Angiopep‐2 functionalization endowed the nanoboron agent with significantly augmented glioma‐targeting specificity. Competitive inhibition assays using free Angiopep‐2 further confirmed that it effectively inhibited the cellular internalization of DM&Ce6@COF‐Ang, confirming that Angiopep‐2 specifically drives internalization via LRP‐1‐mediated RMT (Supporting Information S1: Figure S8). To further quantify the boron accumulation, ICP‐OES was employed to precisely measure the intracellular boron levels at various time points. Consistently, DM@COF‐Ang exhibited significantly higher cellular internalization efficiency than the non‐targeted groups (COF and DM@COF), reaching a boron content of 166.95 ± 11.04 ng per 10^6^ cells at 2 h (Figure 2c). Notably, BPA demonstrated higher internalization efficiency than DM@COF‐Ang in vitro, achieving 211.56 ± 20.16 ng boron per 10^6^ cells at the same time point. This could be explained by the properties of BPA as a near‐neutral and small molecular boron agent that undergoes LAT1‐mediated transport into GL261 cells. In contrast, the poor internalization of BSH is presumably attributable to its anionic nature and high hydrophilicity, which hinder its permeation through the negatively charged phospholipid bilayer of the cell membrane. Subcellular localization of Ce6‐labeled DM@COF‐Ang was further investigated. As shown in Figure 2d, DM&Ce6@COF‐Ang exhibited predominant colocalization with LysoTracker (Pearson's coefficient 0.918) compared to Angiopep‐2 unmodified nanoparticle, DM&Ce6@COF (Pearson's coefficient 0.625), providing compelling evidence that Angiopep‐2 drove the cellular internalization of the nanoboron agent via the RMT. Since the path length of high‐LET particles is confined to the dimensions of a single cell, boron agents distributed in closer proximity to the nucleus are expected to induce more pronounced cellular damage. Thus, as described by Xing et al.,^[12]^ radial lines were extended from the nuclear centroid to segment the cell into three concentric zones, thereby defining the boundaries of three quadrants (Q1–Q3). As shown in Figure 2e,f, approximately 60% of DM&Ce6@COF‐Ang was located in Q2, with a mean distance of 6.26 ± 0.62 μm from the nuclear center. This spatial distribution fell well within the effective range of BNCT.

### 3.3 Blood‐brain barrier (BBB) penetration capability in vitro

To mimic the BBB model in vitro, a Transwell^®^ system was established with bEnd.3 cells in the apical chamber and GL261 cells in the basolateral chamber (Figure 2g). Barrier integrity was confirmed by maintaining a hydrostatic pressure difference >0.6 cm after 5 days of culture (Figure 2h) and by effectively blocking 81.09 ± 1.18% of sodium fluorescein (NaF) within 80 min (Figure 2i). DM&Ce6@COF‐Ang demonstrated significantly enhanced BBB traversal and glioma cell internalization compared to free Ce6 and non‐targeted DM&Ce6@COF (Figure 2j). This result validated the efficacy of Angiopep‐2 functionalization, confirming its role in facilitating RMT across the BBB and enhancing cellular uptake of the nanoboron agent.

### 3.4 Potentiation of anti‐tumor immunity via STING pathway

**FIGURE 3:**
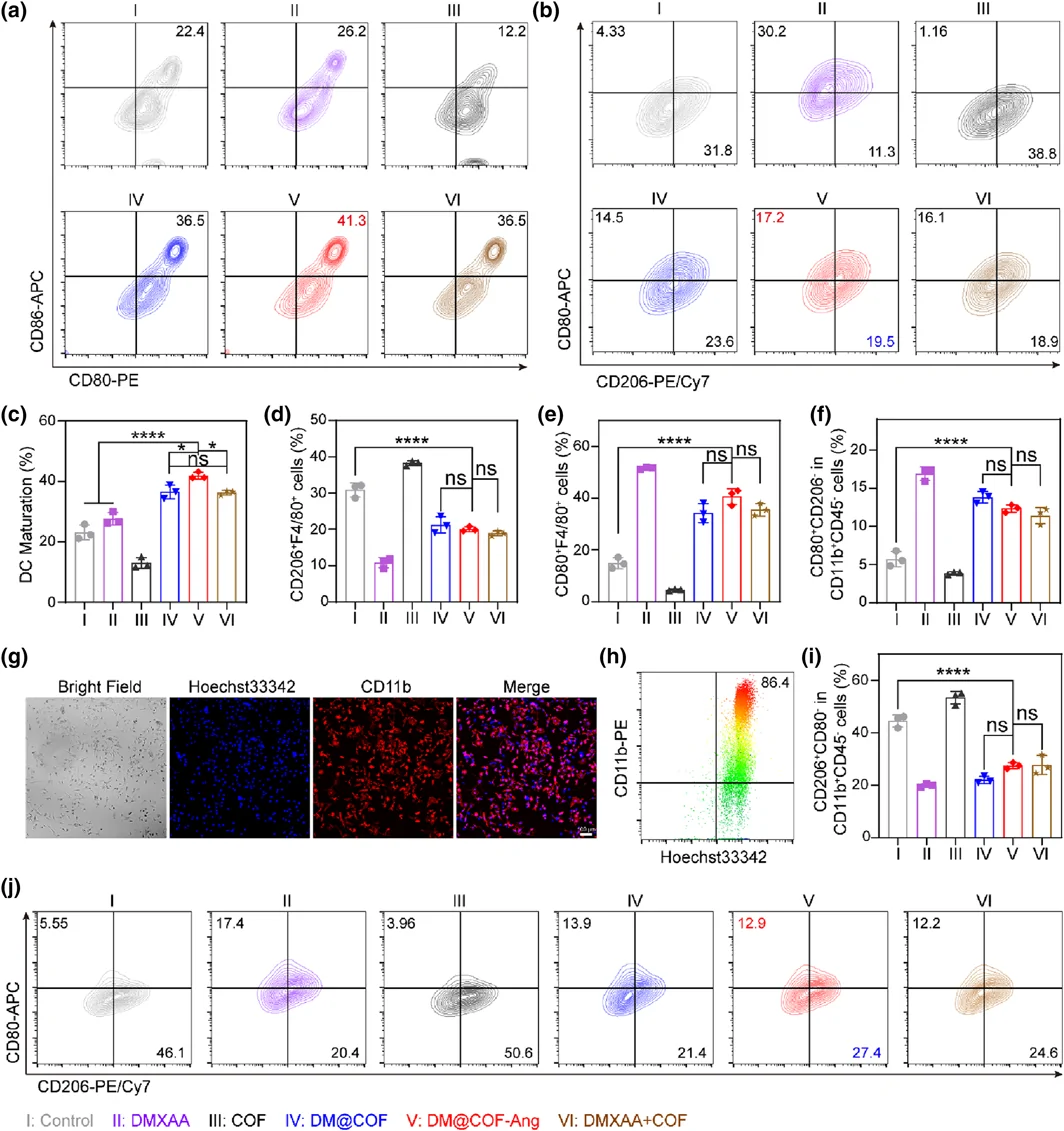
In vitro activation of the STING pathway and enhancement of innate immunity responses. (a, b) Representative of flow cytometric plots of mature BMDCs (CD80^+^CD86^+^ in CD11c^+^ cells) (a) and BMDMs (M1 macrophage: F4/80^+^CD80^+^CD206^‐^, M2 macrophage: F4/80^+^CD80^−^CD206^+^) (b) after different treatments. (c) Quantitative analysis of mature BMDCs after treatment with various conditions (*n* = 3). (d, e) Quantitative analysis of CD80^+^CD206^‐^ M1‐like macrophage (d) and CD206^+^CD80^‐^ M2‐like macrophage (e) by flow cytometry (*n* = 3). (f) Percentages of M1‐like microglia (CD80^+^CD206^−^CD11b^+^CD45^−^) were analyzed by flow cytometry after different treatments (*n* = 3). (g) Representative immunofluorescence images of cultured microglia. Red and blue fluorescence indicated CD11b and nuclei. (h) The purity of cultured microglia was analyzed by flow cytometry. (i) Percentages of M2‐like microglia (CD206^+^CD80^−^CD11b^+^CD45^−^) were analyzed by flow cytometry after different treatments (*n* = 3). (j) Representative of flow cytometric plots of M1‐and M2‐like microglia after different treatments. Data were presented as mean ± SEM. *p*‐values were calculated by using one‐way analysis of variance (ANOVA). *p < 0.05, **p < 0.01, ***p < 0.001, ****p < 0.0001. BMDCs, bone marrow‐derived dendritic cells; BMDMs, bone marrow‐derived macrophages.

DMXAA, a mouse STING agonist, can stimulate immune cells including DCs and reshape the immunosuppressive TME to trigger the host's anti‐tumor immune response.[^55^, ^56^] The activation and maturation of BMDCs was assessed following treatment with PBS, DMXAA, COF, DM@COF, DM@COF‐Ang, or a physical mixture of DMXAA and COF (DMXAA + COF) (Figure 3a,c). Compared with the PBS or COF treated groups, treatment with DM@COF, DM@COF‐Ang and DMXAA + COF significantly promoted BMDC maturation. In addition, the impact on macrophage polarization was examined in IL‐4‐polarized M2‐phenotypic BMM (BMDMs) (Figure 3b,d,e). Biomarker analysis revealed that, compared to the PBS group, DMXAA, DM@COF, DM@COF‐Ang, and DMXAA + COF treatments showed remarkable down‐regulation of CD206 expression (Figure 3d) along with an increase in CD80 expression (Figure 3e). Phenotypic analysis confirmed that DMXAA, DMXAA‐loaded nanoboron agent, and the DMXAA + COF physical mixture all exhibited a comparable ability to repolarize macrophages from an M2 to an M1 phenotype (Figure 3b,d,e). Microglial, the resident innate immune cells of the CNS, are a critical component of inducing immunosuppression in glioma microenvironment.[^57^, ^58^] The purity of cultured microglia was assessed via immunofluorescence and flow cytometry with CD11b and Hoechst 33,342 staining, confirming a purity of 86.4% (Figure 3g,h). The polarization of microglia in response to the various treatments was then investigated. Consistently, DMXAA, DMXAA‐loaded nanoboron agent, and the DMXAA + COF physical mixture demonstrated potent efficacy in reprogramming microglia from the M2‐to M1‐like phenotype (Figure 3f,i,j). The comparable immunomodulatory effects elicited by these three formulations suggested that encapsulation of DMXAA within the nanocarrier did not alter its inherent immunostimulatory activity.

### 3.5 Glioma‐targeting boron delivery and biosafety in vivo

**FIGURE 4:**
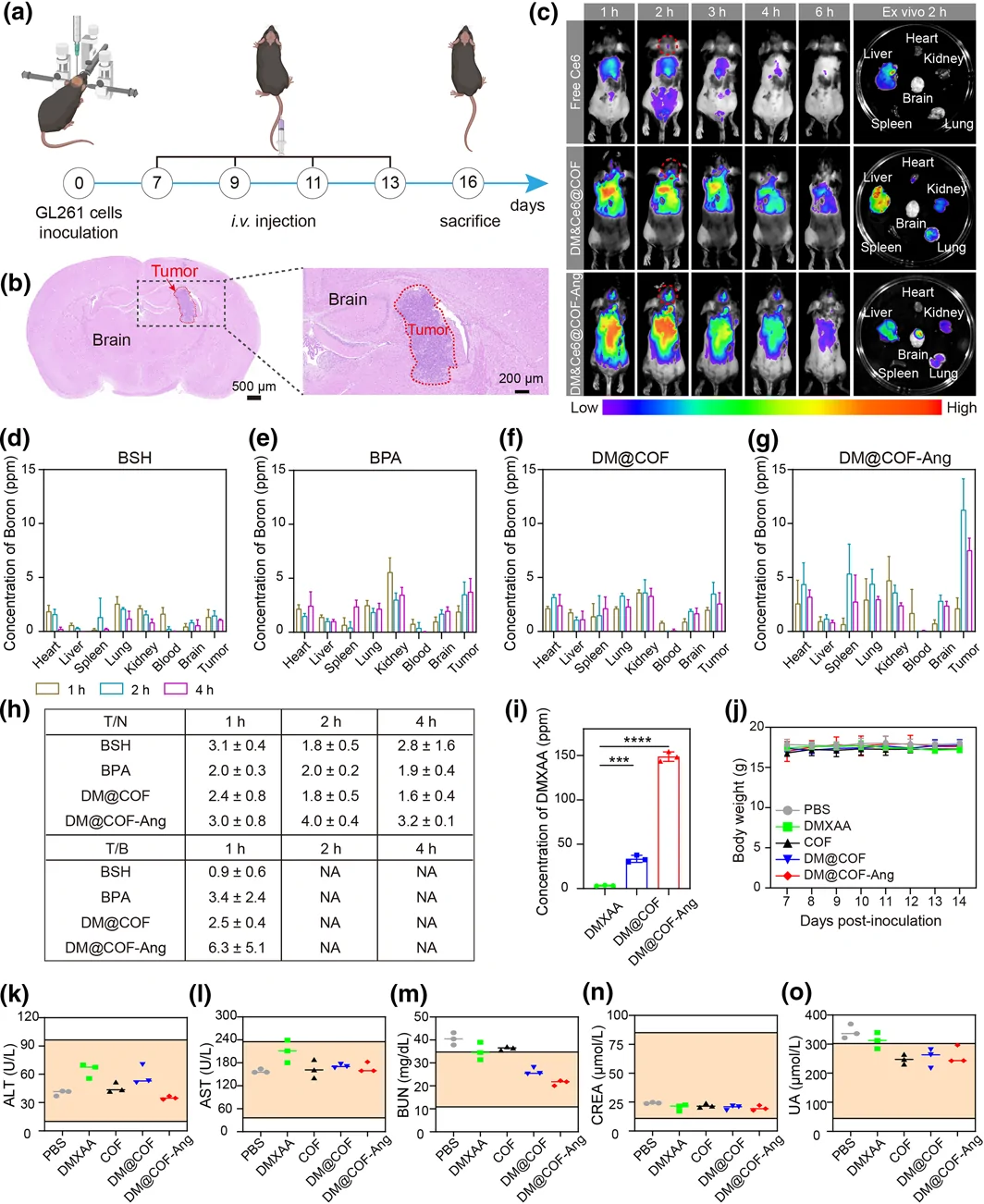
The capacity of nanoboron agent to target orthotopic Glioma and evaluation of biosafety in vivo. (a) The timeline of intracranial inoculation of GL261 cells and treatment courses. (b) Representative H&E staining images (Scale bar, 500 μm) and corresponding enlarged regions (Scale bar, 200 μm) of mouse brains. (c) In vivo real‐time fluorescence imaging of model mice after intravenous injection of free Ce6, DM&Ce6@COF, and DM&Ce6@COF‐Ang at different time points, as well as the ex vivo tissue images after 2 h post‐injection (Ex 665 nm, Em 700 nm). (d–g) Boron concentrations in various organs of mice bearing intracranial glioma at 1, 2, and 4 h after single *i*.*v*. administration of BSH (d), BPA (e), DM@COF (f), and DM@COF‐Ang (g) (3.92 mg B/kg) (*n* = 3 or 4). (h) The tumor to normal brain tissue (T/N) and tumor to blood (T/B) ratios were determined at various time points (1, 2, and 4 h) (*n* = 3 or 4). NA indicated the data were NA. (i) DMXAA concentrations of tumor obtained from mice bearing intracranial glioma at 2 h after single *i*.*v*. administration of DM@COF‐Ang (*n* = 3). (j) Analysis of body weight changes during the 8‐day treatment (*n* = 5). (k–o) Blood biochemistry indexes of GL261 orthotopic glioma mice after administration of PBS, DMXAA, COF, DM@COF, and DM@COF‐Ang, respectively. The levels of alanine aminotransferase (ALT) (k) and aspartate aminotransferase (AST) (l) indicated liver function, and blood urea nitrogen (BUN) (m), creatinine (CREA) (n), and uric acid (UA) (o) indicated renal function (*n* = 3). Data were presented as mean ± SEM. *p*‐values were calculated by using one‐way analysis of variance (ANOVA). *p < 0.05, **p < 0.01, ***p < 0.001, ****p < 0.0001. Ce6, chlorin e6; COF, covalent organic framework; NA, Not Available.

An orthotopic murine glioma model was established in C57BL/6J mice by stereotactically implanting 2 × 10^5^ GL261 cells at coordinates 1 mm anterior, 1 mm lateral (right), and 3 mm deep relative to the bregma (Figure 4a). Histopathological analysis of coronal brain section at 7 days post‐inoculation confirmed successful tumor establishment in the right striatum with distinct lesion formation (Figure 4b). To investigate the tumor‐targeting capability of the nanoboron agent in the orthotopic glioma model and to enable real‐time monitoring of boron delivery agent, tumor‐bearing mice were intravenously injected with free Ce6, the Ce6‐labeled non‐targeted nanoboron agent (DM&Ce6@COF) or the targeted nanoboron agent (DM&Ce6@COF‐Ang), followed by real‐time in vivo fluorescent imaging. The negligible fluorescent signal from Ce6 was detected in the tumor region throughout the monitoring period. Notably, the DM&Ce6@COF‐Ang group exhibited significantly higher tumor fluorescence intensity than the non‐targeted DM&Ce6@COF at all observed time points, with a peak at 2 h post‐injection. The ex vivo image demonstrated predominant distribution of DM&Ce6@COF in other organs such as the liver, lung, and kidney, with minimal signal detected in the brain tumor. In contrast, DM&Ce6@COF‐Ang preferentially accumulated in glioma, with concomitantly reduced uptake in peripheral organs, indicating its profound tumor‐targeting capacity with reduced off‐target effect (Figure 4c).

Next, BSH, BPA, DM@COF, and DM@COF‐Ang at 3.92 mg boron/kg body weight were intravenously injected into GL261 orthotopic glioma mice, respectively. Boron concentrations of heart, liver, spleen, lungs, kidneys, blood, brain, and tumor tissues were measured by ICP‐OES at 1, 2, and 4 h post injection (Figure 4d–g). As expected, the DM@COF‐Ang treatment exhibited superior tumor‐specific boron delivery compared to the non‐targeted DM@COF (3.5 ± 1.0 ppm), achieving 11.3 ± 2.9 ppm in tumor tissue at 2 h post‐injection (Figure 4f,g). This approximately 3.2‐fold increase demonstrated that Angiopep‐2‐mediated transcytosis played a predominant role in augmenting tumor boron accumulation. Given that natural abundance boron contains only ∼19.8% ^10^B, these results validated the delivery efficiency of the nanoplatform as a potential BNCT candidate systemic. In contrast, BSH showed limited and transient tumor accumulation attributable to its rapid systemic clearance, which precluded the maintenance of a therapeutically effective boron concentration in glioma (Figure 4d). Notably, although BPA exhibited higher cellular uptake than DM@COF‐Ang in vitro, its in vivo tumor accumulation was much lower ranging from 1.9 to 3.8 ppm. This discrepancy primarily stemmed from its intrinsic pharmacokinetic profile and limited BBB penetration. While BPA was taken up by GL261 cells via the LAT1 transporter, its efficient delivery to intracranial tumors was substantially impeded by the BBB.[^59^, ^60^, ^61^] Moreover, BPA underwent relatively rapid systemic metabolism and clearance, with renal excretion as the primary elimination pathway (Figure 4e). Furthermore, the tumor to normal brain tissue (T/N) and tumor to blood (T/B) ratios (Figure 4h) were analyzed. Only the DM@COF‐Ang treated group met the clinical requirements of T/N > 3 and T/B > 3. Specifically, it achieved a T/N ratio of 4.0 ± 0.4 at 2 h post‐injection, confirming its efficient glioma delivery of boron in an orthotopic glioma model. In contrast, the T/N ratio for BPA was around 2, a value comparable to the 2.8 reported in another study involving an orthotopic glioma model.^[62]^ Notably, the blood boron concentrations fell below the limit of detection (∼0.1 ppm) of ICP‐OES analysis at 2 h post‐injection. To avoid potentially misleading ratios derived from unreliable measurements, the T/B ratios at the 2–4 h time points were reported as “NA” (Figure 4h). Consistent with this, DM@COF‐Ang treatment also achieved significantly higher levels of DMXAA in tumor region (Figure 4i). Therefore, DM@COF‐Ang demonstrated distinct advantages in terms of both boron and DMXAA enrichment within the glioma.

Next, the in vivo biosafety of the nanoboron agent was assessed by giving a multiple‐dosing regimen. After 7 days of tumor inoculation, the orthotopic glioma mice were randomly assigned to five groups and treated with PBS, DMXAA, COF, DM@COF, and DM@COF‐Ang, respectively (Figure 4a). Throughout the treatment period, the body weights of mice remained consistently stable (Figure 4j), and minimal destructive cell necrosis or inflammation lesions were detected in key organs such as the heart, liver, spleen, lung, and kidney (Supporting Information S1: Figure S9). Furthermore, blood biochemical analysis suggested that treatment with DM@COF‐Ang through the intravenous route had a negligible impact on liver and kidney functions, as indicated by the levels of alanine aminotransferase (ALT), aspartate aminotransferase (AST), blood urea nitrogen, creatinine (CREA), and uric acid (Figure 4k‐o). These data validated the high biocompatibility and safety of DM@COF‐Ang for in vivo applications.

### 3.6 Reprogramming the TME and amplifying CTLs infiltration

**FIGURE 5:**
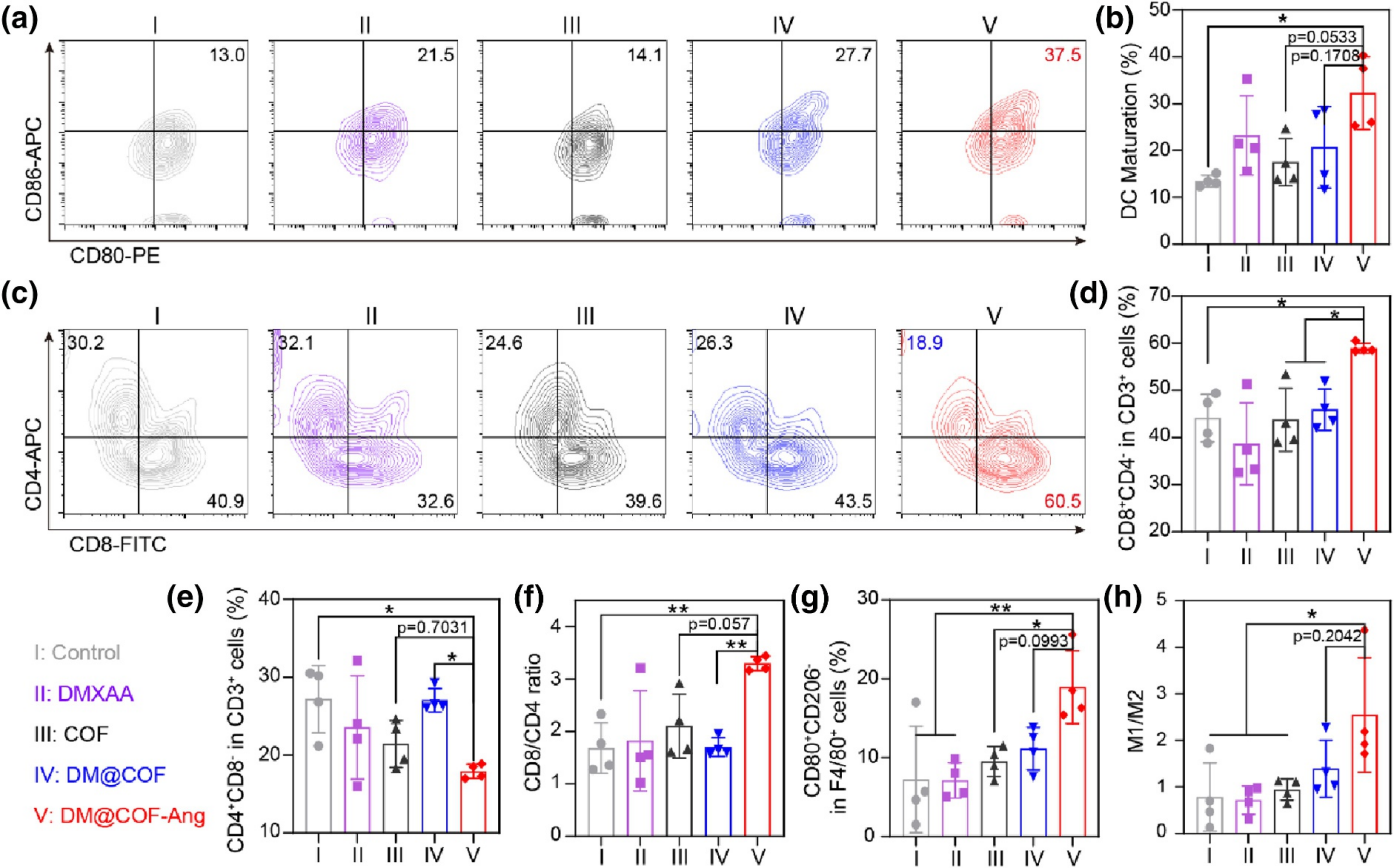
DM@COF‐Ang potentiated the adaptive antitumor immune response in orthotopic glioma model. (a, b) Percentages of mature DCs (CD45^+^CD11c^+^CD80^+^CD86^+^) in gliomas were analyzed by flow cytometry after different treatment, shown as representative dot plots (a) and histograms (b) (*n* = 4). (c) The representative dot plots showed proportions of CD4^+^ and CD8^+^ T cells in the total CD3^+^ cells in brain. (d, e) Number of CD8^+^ (d) and CD4^+^ (e) T cells as a percentage of the total CD3^+^ cells population in glioma by flow cytometry (*n* = 4). (f) The ratio of tumor‐infiltrating CD8^+^/CD4^+^ T cells in each group (*n* = 4). (g) The gliomas were analyzed for CD80^+^CD206^‐^F4/80^+^ M1‐like macrophages measured by flow cytometry (*n* = 4). (h) The amount of the ratio of M1/M2 macrophages in glioma. (*n* = 4). Data were presented as mean ± SEM. *p*‐values were calculated by using one‐way analysis of variance (ANOVA). *p < 0.05, **p < 0.01, ***p < 0.001, ****p < 0.0001.

Glioma is characterized by a highly immunosuppressive and heterogeneous TME with a large number of suppressive immune cells including tumor‐associated macrophages (TAMs).^[63]^ To assess the potential impacts of nanoboron agent on the immunosuppressive TME of glioma, we analyzed the maturation status of DCs, the infiltration of cytotoxic CD8^+^ T lymphocytes (CTLs), and the conversion of TAMs in the orthotopic glioma model. As shown in Figure 5a,b, DM@COF‐Ang treatment (32.3 ± 7.7%) exhibited notable increased levels of DCs activation and maturation (CD11c^+^CD80^+^CD86^+^) in comparison with all other groups (13.5 ± 1.2%, 23.2 ± 8.5%, 17.5 ± 5.0%, 20.7 ± 7.7% for PBS, DMXAA, COF, and DM@COF, respectively) within tumor tissues. DCs are professional APCs that play a crucial role in activating T cell‐mediated antigen‐specific adaptive immune responses.[^64^, ^65^] As a consequence, DM@COF‐Ang treatment led to substantial expansion and infiltration of the CD8^+^ T cell response (Figure 5c,d). Concomitantly, the proportions of CD4^+^ T cells were significantly reduced by DM@COF‐Ang treatment (Figure 5e), which could be a result of the more predominant CD8^+^ T cell response induced by DM@COF‐Ang, and may indicate a concurrent reduction in immunosuppressive regulatory CD4^+^ T cells. Consequently, treatment with DM@COF‐Ang led to an approximate 2‐fold increase in the CD8^+^/CD4^+^ T cell ratio compared to treatment with PBS (Figure 5f). Further, the phenotypic of TAMs induced by nanoboron agent was determined. As illustrated in Figure 5g, DM@COF‐Ang induced increases of 2.62‐, 2.66‐, 1.99‐, 1.70‐fold in M1‐phenotypic (CD80^+^CD206^−^F4/80^+^) macrophages compared with the PBS, DMXAA, COF, and DM@COF groups, respectively. This was accompanied by a significant elevation in the M1/M2 phenotypic ratio (Figure 5h), which was primarily attributed to the enhanced accumulation of DMXAA in glioma facilitated by DM@COF‐Ang. All these results demonstrated that DM@COF‐Ang favorably altered the non‐immunogenic “cold” tumors into immunogenic “hot” ones.

### 3.7 Boosting the systemic anti‐tumor immunity

**FIGURE 6:**
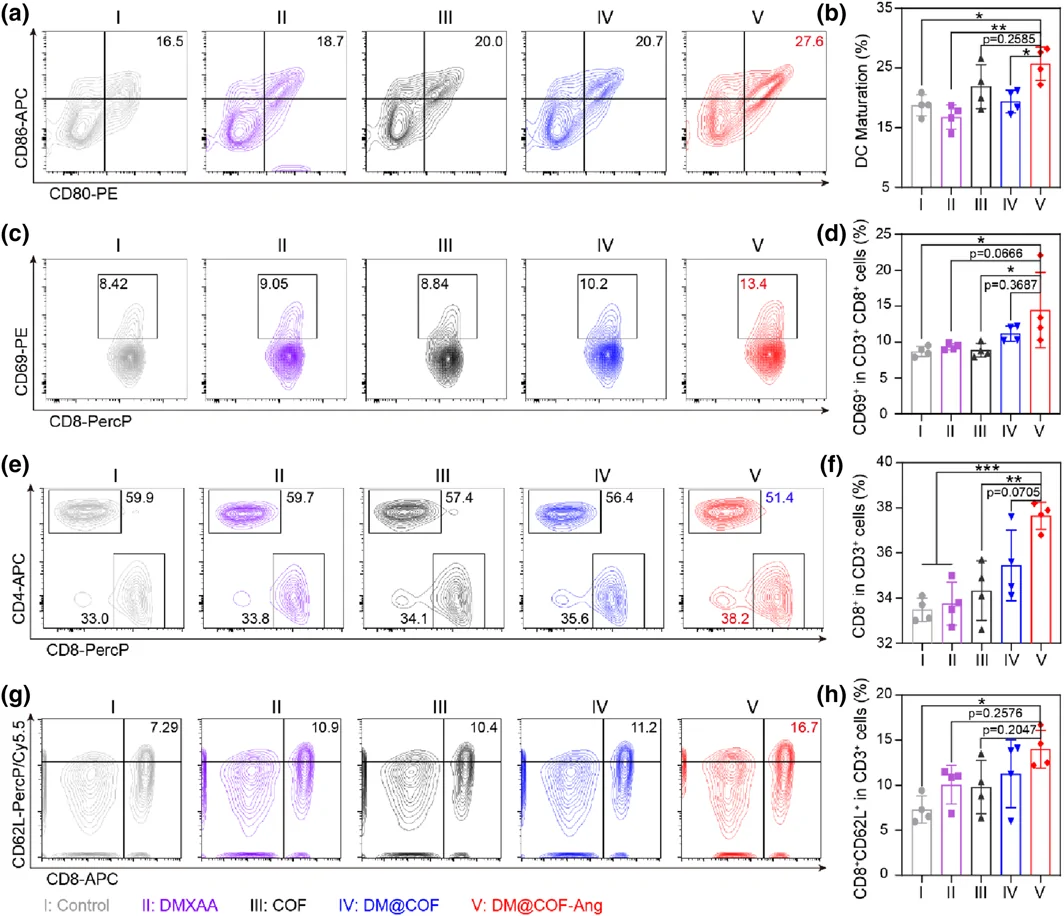
DM@COF‐Ang potentiated the systemic immune response in orthotopic glioma model. (a, b) Representative dot plots (a) and histograms (b) (*n* = 4) of mature DCs (CD11c^+^CD80^+^CD86^+^) in tumor‐draining lymph nodes (TDLNs). (c, d) Representative dot plots (c) and histograms (d) (*n* = 4) of CD69^+^CD8^+^ T cells after gating on CD3^+^ cells in TDLNs. (e, f) Splenocytes from different treated groups were analyzed for percentage of CD8^+^ T cells in the total CD3^+^ cell population, shown as representative dot plots (e) and histograms (f) (*n* = 4). (g, h) Representative flow cytometry plots (g) and histograms (h) (*n* = 4) of CD62L^+^CD8^+^ memory T cells in CD3^+^ cells in the spleens. Data were presented as mean ± SEM. *p*‐values were calculated by using one‐way analysis of variance (ANOVA). *p < 0.05, **p < 0.01, ***p < 0.001, ****p < 0.0001.

Tumor‐draining lymph nodes are crucial peripheral lymphoid organs where mature DCs cross‐present tumor antigens to the T lymphocytes to induce systemic anti‐tumor immunity.^[66]^ To examine the activated status and cross‐presentation of DCs, TDLNs were collected and analyzed via flow cytometry. As shown in Figure 6a,b, mice treated with DM@COF‐Ang exhibited a remarkable enrichment of mature DCs (CD80^+^CD86^+^CD11c^+^) in TDLNs. Specifically, the proportions of activated DCs in TDLNs increased by 1.37‐, 1.53‐, 1.17‐, and 1.33‐fold relative to the PBS, DMXAA, COF, and DM@COF groups, respectively. Meanwhile, the significant activation and expansion of CD8^+^ T cells in the DM@COF‐Ang treated group were observed (Figure 6c,d). We next analyzed the systemic adoptive anti‐tumor immune responses. Remarkably increased splenic CD8^+^ T cells (37.56 ± 0.60%) were observed following DM@COF‐Ang treatment, which was about 1.1‐fold higher compared to all other groups (Figure 6e,f). More importantly, a higher number of CD8^+^ T cells had been differentiated into memory phenotypes (Figure 6g,h), indicating an enhanced capacity for anti‐tumor immune protection. These findings demonstrated that DM@COF‐Ang was capable of triggering robust and long‐lasting systemic anti‐tumor immune responses against well‐established glioma.

## 4 DISCUSSION AND CONCLUSION

Efficient boron delivery is a prerequisite for successful BNCT.^[67]^ Although BPA remains the most widely used boron agent due to its selective accumulation in tumors via amino acid transporters, LAT1, its clinical efficacy, particularly for glioma, is severely compromised by rapid systemic clearance and poor BBB penetration.[^20^, ^21^] In this work, we developed an Angiopep‐2 functionalized, glioma‐targeting multifunctional nanoboron agent (DM&Ce6@COF‐Ang) using APTES‐COF‐1 with a high inherent boron content (13.5%), as both the potential therapeutic boron agent and the nanocarrier.

In an orthotopic glioma‐bearing murine model, DM@COF‐Ang demonstrated superior tumor targeting capacity, with a T/N ratio of 4.0 ± 0.4 at 2 h post‐injection, substantially exceeding those of BPA treated group (T/N ≈ 2). Nevertheless, the peak tumor boron concentration by DM@COF‐Ang treatment was 11.3 ± 2.9 ppm, which falls below the conventional threshold (20 ppm) for ideal boron delivery agents. A substantially higher tumor boron level of ∼67.5 ppm was reported in a previous study using zirconium meso‐tetra(4‐carboxyphenyl)porphyrin (Zr‐TCPP) metal‐organic frameworks as the boron nanocarrier; however, that result was achieved at an injection dose of 50 mg boron/kg.^[68]^ In stark contrast, the DM@COF‐Ang treatment achieved a tumor boron level of 11.3 ± 2.9 ppm at a dose of only 3.92 mg boron/kg, less than one‐10th of 50 mg boron/kg, underscoring both the high boron delivery efficiency of the nanoboron agent and the substantial therapeutic window for dose escalation. Importantly, the conservative boron dose (3.92 mg/kg) used in our study was dictated by the co‐delivered immunomodulator DMXAA at 15 mg/kg, which lies within the conventional dosage range (8–15 mg/kg) applied in the most published studies.[^69^, ^70^, ^71^, ^72^] Future optimization strategies will focus on safely elevating the boron levels to meet the 20 ppm threshold. First, reducing the DMXAA‐to‐APTES‐COF‐1 loading ratio in the nanoboron formulation would allow for a higher single‐injection dose of APTES‐COF‐1 while maintaining DMXAA within a safe and effective range. Second, intratumoral boron accumulation could be enhanced through a multiple‐injection regimen. The feasibility of this approach is supported by prior reports.[^23^, ^73^, ^74^] One study demonstrated that three injections produced significantly higher (1.54‐fold) boron concentration at the tumor site compared with a single injection, along with improved tumor to normal tissue (T/N) and tumor to blood (T/B) ratios.^[74]^ Meanwhile, the APTES‐COF‐1 utilized in this study comprised boron at natural isotopic abundance, wherein the therapeutic ^10^B isotope accounts for only 19.8% of total boron content.[^75^, ^76^] The 11.3 ppm natural abundance of boron corresponded to merely 2.24 ppm therapeutic ^10^B. Eventually, boron isotopes (^10^B and ^11^B) exhibit virtually similar absorption, distribution, metabolism, and excretion (ADME) profiles in vivo, owing to their identical chemical properties.^[77]^ Therefore, the natural abundance of boron was utilized as a cost‐effective surrogate to validate the delivery efficiency of the nanoplatform rather than its BNCT therapeutic efficacy. Consequently, the total boron payload achieved by the nanoplatform in this study provides a reliable quantitative benchmark for predicting the therapeutic efficacy of a future ^10^B‐enriched formulation. Specifically, a ^10^B‐enriched boron precursor (^10^B‐BDBA) is required for future thermal neutron irradiation to rigorously evaluate the therapeutic outcome of the nanoboron agent‐mediated BNCT in combination with immunotherapy. Although the targeting efficacy of DM@COF‐Ang in vivo has been established, it is imperative to underscore the validity of the in vitro BBB model employed in this study. The BBB integrity and tightness were validated by trans‐endothelial hydraulic conductivity and sodium fluorescein permeability assays.

Real‐time monitoring of boron agents is crucial in BNCT as tumor heterogeneity drives marked inter‐patient variability in the pharmacokinetic profiles of even the same boron agent, thereby compromising therapeutic outcomes.^[80]^ COFs have emerged as versatile nanocarriers for various imaging agents owing to their high specific surface area and tunable pore size.[^25^, ^74^] In this work, Ce6 was employed as a model imaging agent and demonstrated the ability to effectively probe the biodistribution of the nanoboron agent. Nevertheless, the accuracy of fluorescence imaging is inferior to that of the gold‐standard clinical molecular imaging modality, ^18^F‐labeled PET/computed tomography (CT).^[81]^ Beyond fluorescence imaging, COFs are also capable of delivering radionuclide‐based contrast agents for PET/CT. The expanding molecular imaging landscape, including magnetic resonance imaging, can be readily integrated into COF platforms as well.^[82]^ Leveraging modular design, COF‐based nanoplatforms could be accommodated with diverse imaging agents for multimodal detection, broadening the visualization toolkit for precision BNCT.

The immunosuppressive TME in glioma is known to significantly hamper BNCT‐mediated anti‐tumor immunity.[^1^, ^49^, ^50^] In this study, we investigated whether co‐delivery of the STING agonist DMXAA could reprogram this immunosuppressive milieu. Our results demonstrated that DMXAA elicited pronounced immunomodulatory effects within an orthotopic murine glioma model, including DC maturation, macrophage polarization toward an anti‐tumor phenotype, and enhanced CD8+ T cell activation and infiltration. These findings collectively indicated the establishment of a pre‐conditioned, immunostimulatory TME that holds the potential to potentiate the therapeutic outcome of subsequent BNCT. Nevertheless, direct experimental evidence for synergistic action between DMXAA‐mediated immunomodulation and BNCT remains to be established via thermal neutron irradiation studies. Furthermore, although DMXAA is effective in murine models, it exhibits minimal activity in humans.[^56^, ^83^] Of note, DMXAA served as a model immune adjuvant and was incorporated into the nanoboron agent via hydrophobic interactions without compromising its immunostimulatory activity. In subsequent studies, the model adjuvant DMXAA can be substituted by hydrophobic immune adjuvants, such as MSA‐2, a human STING agonist and imiquimod, a human TLR7/8 agonist. The modularity of this nanoplatform permits the selection of appropriate immunoadjuvants based on individual patient, laying the groundwork for future personalized BNCT‐immunotherapy combinations.

In summary, we developed a theranostic strategy for glioma by engineering a multifunctional, brain‐targeting nanoplatform (DM&Ce6@COF‐Ang). The integrated nanoplatform was constructed by functionalizing a boron‐embedded APTES‐COF‐1 with the targeting ligand Angiopep‐2, co‐delivering the immune modulator, DMXAA as well as the fluorescent probe, Ce6. This design established a versatile nanoplatform that concurrently addressed the key challenges of tumor‐selective delivery, real‐time monitoring, and immunomodulation for glioma. Importantly, this modular strategy provides a robust foundation for the future development of personalized BNCT regimens through the adaptable co‐delivery of diverse therapeutic molecules and imaging agents.

## CONFLICT OF INTEREST STATEMENT

The authors declare no conflicts of interest.

## ETHICS STATEMENT

All animal experimental protocols were approved by the Biological and Medical Ethics Committee of Dalian University of Technology (approval number: DUTSCE260403‐01), and complied with the rules of the specific pathogen‐free animal laboratory. All methods followed the guidelines for animal subject care and use outlined in the Guide for the Care and Use of Laboratory Animals (Institute of Laboratory Animals Resources, National Academy of Sciences, Bethesda, MD, USA).

## Supporting information

Supporting Information S1

## Data Availability

The data that support the findings of this study are available from the corresponding author upon reasonable request.
