# Supplementary material for: Boron‐embedded multifunctional nanoplatform toward potential combined boron neutron capture therapy and immunomodulation in glioma
Source: Smart Mol. 2026 Sep 16:e70107. Online ahead of print. doi: 10.1002/smo2.70107 (PMC13580511; doi:10.1002/smo2.70107)
Supplement: Supplementary file 1 [file SMO2-9999-0-s001.docx]

Boron-embedded multifunctional nanoplatform towards potential combined boron neutron capture therapy and immunomodulation in glioma

Yiming Liu^1, 2#^, Xuejian Wang^3#^, Bangjian Li^2^, Kun Shao^2^*, Jianlong Su^2^, Yang Wang^2^, Lin Cheng^1^, Zhihao Wu^2^, JinRong Li^2^, Guanyi Zhang^2^, Xu Wang^2^, Yuxuan Zhang^2^, Yuanhao Liu^4^, Guangzhe Li^1^*

^1^ State Key Laboratory of Fine Chemicals, Department of Pharmaceutical Engineering, School of Chemical Engineering, Dalian University of Technology, Dalian 116024, China.

^2^ State Key Laboratory of Fine Chemicals, Frontiers Science Center for Smart Materials Oriented Chemical Engineering, School of Chemical Engineering, Dalian University of Technology, Dalian 116024, China.

^3^ Department of Urology, First Affiliated Hospital of Dalian Medical University, Dalian, 116021, China.

^4^ Neuboron Medtech Ltd., Nanjing 211112, China.

*Corresponding authors. Email: shaok@dlut.edu.cn (K. S.); liguangzhe@dlut.edu.cn (G. L.)

^#^Yiming Liu and Xuejian Wang contributed equally to this work.

**Supplementary Figures**

**Figure. S1.** ^11^B multiple quantum magic angle spinning (MQMAS) NMR spectra of APTES-COF-1.

**Figure. S2.** Reaction kinetics monitoring of DSPE-PEG_2000_-Mal and Cys-Angiopep-2.

**Figure. S3.** ^1^H NMR (600 MHz, DMSO) of DSPE-PEG_2k_-Ang and DSPE-PEG_2k_-Mal.

**Figure. S4.** The characteristic Ce6 UV peak in each tube after dextran gel chromatography of unpurified and purified nanoboron agent.

**Figure. S5.** AFM imaging of DM&Ce6@COF-Ang nanoboron agent.

**Figure. S6.** Time-dependent boron release profiles of BSH and DM&Ce6@COF-Ang nanoboron agent in PBS supplemented with 56% FBS (Mean ± S.D., n = 3). The boron level was quantified by ICP-OES.

**Figure. S7.** Live cell imaging of bEND.3 and GL261 cells demonstrating the cellular uptake of Ce6, DM&Ce6@COF and DM&Ce6@COF-Ang at different time points. The nuclei of cells were stained with DAPI.

**Figure. S8.** (**a**) Representative fluorescence micrographs and (**b**) mean fluorescence intensity (MFI) of GL261 cells analyzed by flow cytometry showing the competitive inhibitory internalization of DM&Ce6@COF-Ang (5 μg/mL of Ce6) with Angiopep-2 blockade (10 μg/mL) at 2 h. The nuclei of cells were stained with DAPI. Scale bar, 100 μm. Data were presented as mean ± SEM. P-values were calculated by using student’s t test and one-way analysis of variance (ANOVA). *P < 0.05, **P < 0.01, ***P < 0.001, ****P < 0.0001.

**Figure. S9.** H&E staining of major organs (heart, liver, spleen, lung, and kidney) of differently treated mice for *in vivo* biosafety evaluation. Scale bar, 100 μm.
